# Higher Adherence to the Mediterranean Dietary Pattern Is Inversely Associated With Severity of COVID-19 and Related Symptoms: A Cross-Sectional Study

**DOI:** 10.3389/fmed.2022.911273

**Published:** 2022-07-19

**Authors:** Nikan Zargarzadeh, Kiana Tadbir Vajargah, Armin Ebrahimzadeh, Seyed Mohammad Mousavi, Hamidreza Khodaveisi, Camellia Akhgarjand, Fernando M. P. Toyos, Henrique S. Cerqueira, Heitor O. Santos, Mohsen Taghizadeh, Alireza Milajerdi

**Affiliations:** ^1^School of Medicine, Tehran University of Medical Sciences, Tehran, Iran; ^2^Research Center for Biochemistry and Nutrition in Metabolic Diseases, Institute for Basic Sciences, Kashan University of Medical Sciences, Kashan, Iran; ^3^Obesity and Eating Habits Research Center, Endocrinology and Metabolism Clinical Sciences Institute, Tehran University of Medical Sciences, Tehran, Iran; ^4^Autoimmune Bullous Diseases Research Center, Razi Hospital, Tehran University of Medical Sciences, Tehran, Iran; ^5^Department of Clinical Nutrition, School of Nutritional Sciences and Dietetics, Tehran University of Medical Sciences, Tehran, Iran; ^6^Postgraduate Program, UNIGUAÇU, Porto União, Brazil; ^7^School of Medicine, University of São Paulo, São Paulo, Brazil; ^8^School of Medicine, Federal University of Uberlandia (UFU), Uberlandia, Brazil

**Keywords:** COVID-19, dietary pattern, severe disease, infectious disease, Mediterranean diet

## Abstract

**Background and Aims:**

Adherence to the Mediterranean diet (MD) has been associated with a decreased risk of developing a variety of chronic diseases that are comorbidities in COVID-19 patients. However, its association to the severity and symptoms of COVID-19 are still unknown. This study aimed to examine the association between adherence to the MD pattern and COVID-19 severity and symptoms in Iranian hospitalized patients.

**Methods:**

In this cross-sectional study, 250 COVID-19 patients aged 18 to 65 were examined. We employed a food frequency questionnaire (FFQ) to obtain data on dietary intake of participants in the year prior to their COVID-19 diagnosis. COVID-19 severity was determined using the National Institutes of Health's Coronavirus Disease 2019 report. Additionally, symptoms associated with COVID-19, inflammatory markers, and other variables were evaluated. The scoring method proposed by Trichopoulou et al. was used to assess adherence to the MD.

**Results:**

The participants' mean age was 44.1 ± 12.1 years, and 46% of them had severe COVID-19. Patients who adhered more closely to the MD had lower serum C-reactive protein levels (7.80 vs. 37.36 mg/l) and erythrocyte sedimentation rate (14.08 vs. 42.65 mm/h). Those with the highest MD score were 77% less likely to have severe COVID-19 after controlling for confounding variables. The MD score was also found to be inversely associated with COVID-19 symptoms, including dyspnea, cough, fever, chills, weakness, myalgia, nausea and vomiting, and sore throat.

**Conclusion:**

Higher adherence to the MD was associated with a decreased likelihood of COVID-19 severity and symptoms, as well as a shorter duration of hospitalization and convalescence, and inflammatory biomarkers.

## Introduction

Coronavirus disease 2019 (COVID-19), a viral illness caused by severe acute respiratory syndrome coronavirus 2 (SARS-CoV-2), continues to be a global health concern, resulting in over six million deaths worldwide by the end of March 2022 (1). It is no surprise that this has become the pandemic the most impacting pandemic of this century (2, 3). The COVID-19 pandemic has profoundly affected numerous facets of global life, including health care systems and the economy (4, 5). COVID-19, in addition to causing significant global management disorders, can result in aggressive sequelae and death as a result of a harmful link between the immune, respiratory, and cardiovascular systems (6–8). Besides advanced pharmacological treatments, non-pharmacological interventions have gained attention to assist in combating primary and secondary events of COVID-19 (9, 10). In this regard, dietary habits are critical due to their long-term physiological necessity (11).

While COVID-19 is a disease with a strong inflammatory stimulus that manifests primarily in moderate to severe conditions, resulting in a “cytokine storm” as a result of an excessive immune response (12, 13), numerous nutraceutical agents (e.g., herbal medicines), personalized vitamin and mineral dosing regimens, and dietary patterns all have anti-inflammatory properties that appear to reduce the production and action of inflammatory molecules and pathways (14–16). Recent randomized clinical trials (RCTs) showed that nutrient supplementation alone has no discernible effect on the management of COVID-19 and should be used only as a supplement to correct a diagnosed nutritional deficiency (17, 18). Dietary patterns and COVID-19 have received less attention, with most studies on dietary patterns focusing on dietary changes caused by the pandemic or lockdown (19). Interestingly, following plant-based diets were associated with a decreased risk of moderate-to-severe COVID-19 infection in a population-based case–control study involving 2,884 participants from six countries (20). In addition, an observational study of 509 COVID-19 patients suggested an inverse association between adherence to a vegetarian diet and severity of COVID-19 symptoms as well as an increased risk for non-vegetarians (21).

The Mediterranean diet (MD) is a widespread dietary pattern associated with anti-inflammatory properties due to its high consumption of olive oil, nuts, seeds, vegetables, fruits, whole grains, low-fat dairy, and low intake of meats and dairy products (22–25). The MD is a healthy dietary approach that promotes overall well-being (26) and is associated with risk reduction of the common comorbidities observed in COVID-19 patients (27). Indeed, MD patterns have a preventive effect on cardiovascular diseases and diabetes, which are risk factors for severe COVID-19 infection and its associated complications (28). Thus, the present study aimed to investigate the association between adherence to the MD pattern and the severity of COVID-19 and related symptoms in a sample of adult Iranian hospitalized patients.

## Methods

### Participants

We conducted a retrospective cross-sectional study on 250 patients aged 18–65 who had recovered from COVID-19. Using a convenience sampling method, participants were drawn from Shahid Beheshti Hospital in Kashan, Iran. This research was conducted between June and September 2021. The Kashan University of Medical Sciences Ethics Committee reviewed and approved the study's protocol (Registration No. IR.KAUMS.MEDNT.REC.1400.048). Informed consent was obtained from study participants prior to enrollment in the study.

At first, we examined the medical records of 600 COVID-19 patients with available medical data from Shahid Beheshti Hospital who had been diagnosed within the previous 3 months were included. We excluded 350 patients after considering the following items as the exclusion criteria: any comorbidities or diseases other than COVID-19, such as diabetes, cardiovascular disease, or diseases that affect COVID-19 severity; a body mass index (BMI) >40 kg/m^2^; current pregnancy or breastfeeding; active smoking; consuming dietary supplements more than twice a week prior to the COVID-19 diagnosis; being on any special diets; taking any medications that affect respiratory tract function, such as fluticasone, flunisolide, and so on; and incomplete medical records. In addition, all enrolled patients were not vaccinated because public vaccination had not yet begun in Iran at the time of the study.

### Assessment of Dietary Intakes

Dietary intakes were assessed using a web-based 168-item food frequency questionnaire (FFQ) to collect data on dietary intakes of participants during a year preceding their COVID-19 diagnosis. The reliability and validity of the FFQ have previously been evaluated (29). We utilized the FFQ to assess dietary intakes because the patients in this study were hospitalized and their diets may have changed due to the disease and its consequences. This questionnaire allowed participants to report their dietary intakes daily, monthly, or annually. Then, using 'household measures,' the dietary intakes were converted to grams per day (g/d). Nutritionist IV software was used to calculate the dietary intakes of micronutrients and macronutrients.

Adherence to the MD was used following the method introduced by Trichopoulou et al. (30). The adherence was quantitated by calculating the MD score. To accomplish this, nine components were taken into account: vegetables, fruits, nuts, legumes, fish, whole grains, the ratio of monounsaturated fatty acids (MUFAs) to saturated fatty acids (SFAs), meats (red meat, poultry, and processed meats), and dairy. Participants who were above the median intakes of vegetables, fruits, nuts, legumes, fish, whole grains, and the highest MUFA to SFA ratio and those who were below the median intakes of meats and dairy received a score of 1. In contrast, individuals at the top median intakes of meat and dairy products as well as those at the bottom median intakes of whole grains, fruits, vegetables, fish, nuts, and legumes received a score of 0. These scores were summed up to calculate the overall MD score.

### Measurement of COVID-19 Severity

The severity of COVID-19 was described and classified using the National Institute of Health's Coronavirus Disease 2019 (COVID-19) Treatment Guidelines, updated on October 19, 2021 (31). This guideline defines patients with severe illness as those who have SpO_2_ <94% on room air at sea level, arterial partial oxygen pressure/fraction of inspired oxygen (PaO_2_/FiO_2_) <300 mmHg, respiratory rate (RR) >30 breaths/min, or lung infiltrates >50%), and a cluster of respiratory failure, septic shock, and/or multiple organ dysfunction.

### Measurement of COVID-19 Symptoms

Patients were asked to fill out a questionnaire including questions about the presence of common COVID-19 manifestations, i.e., fever, chilling, dyspnea, cough, weakness, muscle pain, sore throat, nausea, and vomiting.

### Assessment of Inflammatory Markers

The first available measures of erythrocyte sedimentation rate (ESR) and C-reactive protein (CRP) were obtained from medical records during patients' hospitalization.

### Assessment of Other Variables

A general questionnaire was used to collect information about each patient's demographics, physical activity, convalescence duration, administration of supplement, corticosteroid, and antiviral drug intake, and participants' height and weight.

### Statistical Analysis

We divided participants into tertile groups based on their MD score. To determine normality, we used the Kolmogorov-Smirnov test. Means and standard deviations for continuous variables and percentages for categorical variables were reported to describe the general characteristics of participants across tertiles of MD score. The general characteristics of study participants were compared using one-way analysis of variance (ANOVA) for continuous and chi-square tests for quantitative variables across tertiles of MD score. The inflammatory biomarkers were compared using covariance analysis (ANCOVA) across the MD score tertiles, after adjustment for age, gender, BMI, and physical activity. The association between the MD score and severe COVID-19, as well as its symptoms was assessed using binary logistic regression in multiple models. At the first model findings were adjusted for age (continuous), gender (male/female), and energy intake/BMR (continuous). In the second model, additional adjustments were made for (sedentary/moderate/intense), supplementation (yes/no), corticosteroid use (yes/no), and antiviral medication use (yes/no). In the third model, there was an additional adjustment for the BMI (continuous). The Statistical Package for the Social Sciences (SPSS Inc., version 25) was used in the current study. Statistical significance was defined as *P*-values <0.05.

## Results

The general characteristics of study participants across tertiles of MD score are summarized in Table 1. The mean age and gender distribution were not significantly different across the MD score tertiles. Participants at the highest tertile of MD score had a lower BMI and were less likely to be overweight or obese. Furthermore, they had a significantly shorter length of hospitalization and convalescence than those in the lowest tertile.

**Table 1:** General characteristics of participants across tertiles of Mediterranean diet.

	**Tertiles of MD score**			
	**T1**	**T2**	**T3**	P^*^
	***n = 80***	***n = 81***	***n = 89***	
Age (years)	44.7 ± 12.6	41.9 ± 12.0	45.7 ± 11.7	0.12
Female (%)	61.3	50.6	46.1	0.13
Body mass index (kg/m^2^)	29.3 ± 3.7	26.1 ± 3.5	25.7 ± 3.0	<0.001
Physical activity
Sedentary	18.8	7.4	11.2	0.12
Moderate	73.8	88.9	79.8	
Intense	7.5	3.7	9.0	
Overweight or obese (%)	88.8	61.7	52.8	<0.001
Supplements intake (%)	98.8	91.4	94.4	0.10
Corticosteroids use (%)	97.5	87.7	91.0	0.06
Antiviral Drugs use (%)	97.5	87.7	91.0	0.06
Duration of hospitalization (d)	8.1 ± 3.2	5.7 ± 2.4	5.9 ± 2.6	<0.001
Convalescence duration (d)	10.8 ± 3.8	9.5 ± 4.1	8.2 ± 2.9	<0.001

* *Obtained from ANOVA or Chi-square test, where appropriate*.

Table 2 depicts dietary intakes of selected food groups and nutrients across tertiles of the MD score. Compared to the lowest tertile, participants at the top tertile of the MD score had higher intakes of protein, carbohydrate, dietary fiber, n-3 fatty acids, vitamins B1, B2, B6, B12, folate, calcium potassium, magnesium intakes when compared to those at the lowest tertile. These participants also had lower daily energy, fat, SFAs, MUFAs, vitamin B3, and vitamin D intakes. It is worth noting that those at the highest tertile of the MD score significantly consumed more fruits, vegetables, legumes, nuts, whole grains, and fish and lower meats than those at the.

**Table 2:** Selected food groups and nutrients intakes of participants across tertiles of Mediterranean diet score.

	**Tertiles of MD score**			
	**T1**	**T2**	**T3**	***P* ^a^**
	***n = 80***	***n = 81***	***n = 89***	
**Nutrients**
Energy (kcal/d)	2,811.4 ± 552.7	2,638.3 ± 470.3	2,788.6 ± 351.2	0.04
Protein (g/d)	101.3 ± 15.7	108.1 ± 19.1	114.2 ± 14.0	<0.001
Fat (g/d)	109.8 ± 33.1	97.0 ± 26.3	96.3 ± 15.9	0.005
Carbohydrate (g/d)	416.6 ± 58.9	393.0 ± 55.3	419.6 ± 43.5	0.002
Cholesterol (mg/d)	432.5 ± 146.7	450.8 ± 160.1	448.2 ± 161.0	0.72
Dietary fiber (g/d)	18.7 ± 2.7	23.2 ± 4.0	27.1 ± 2.9	<0.001
SFAs (g/d)	34.2 ± 11.6	27.3 ± 9.5	25.0 ± 4.9	<0.001
MUFAs (g/d)	34.3 ± 11.3	27.5 ± 8.7	27.7 ± 6.1	<0.001
n-3 Fatty acids (g/d)	0.33 ± 0.11	0.34 ± 0.16	0.52 ± 0.15	<0.001
Vitamin B1 (mg/d)	2.5 ± 0.3	2.4 ± 0.4	2.6 ± 0.3	0.001
Vitamin B2 (mg/d)	1.8 ± 0.3	2.0 ± 0.4	2.1 ± 0.3	<0.001
Vitamin B3 (mg/d)	28.2 ± 4.4	26.5 ± 4.5	27.5 ± 3.4	0.04
Vitamin B6 (mg/d)	1.5 ± 0.3	1.7 ± 0.3	1.9 ± 0.2	<0.001
Folate (μg/d)	337.5 ± 61.7	427.6 ± 80.1	476.6 ± 65.7	<0.001
Vitamin B12 (μg/d)	3.6 ± 0.9	4.0 ± 1.4	4.9 ± 1.1	<0.001
Vitamin D (μg/d)	2.53 ± 0.90	2.21 ± 0.68	2.11 ± 0.56	0.003
Calcium (mg/d)	843.8 ± 125.9	917.6 ± 140.0	961.5 ± 106.8	<0.001
Potassium (mg/d)	3,274.3 ± 488.1	3,703.4 ± 520.8	4,136.0 ± 425.9	<0.001
Magnesium (mg/d)	292.0 ± 43.3	326.5 ± 49.6	364.3 ± 39.1	<0.001
**Food groups**
Fruits (g/d)	241.0 ± 66.7	368.4 ± 109.1	448.0 ± 69.7	<0.001
Vegetables (g/d)	196.8 ± 46.0	286.8 ± 92.1	342.0 ± 79.6	<0.001
Whole grains (g/d)	49.4 ± 61.4	116.2 ± 84.3	81.8 ± 76.1	<0.001
Legumes (g/d)	92.6 ± 22.0	143.2 ± 37.1	163.3 ± 32.6	<0.001
Nuts (g/d)	19.9 ± 9.8	30.8 ± 13.0	39.5 ± 10.3	<0.001
Fish (g/d)	12.8 ± 6.1	20.6 ± 11.9	34.2 ± 10.7	<0.001
Dairy (g/d)	278.3 ± 82.9	295.0 ± 69.7	291.9 ± 48.2	0.35
Meats and meat products (g/d)	119.4 ± 34.4	106.5 ± 29.8	96.9 ± 30.8	<0.001

*MUFAs, monounsaturated fatty acids; SFAs, saturated fatty acids*.

*Data are presented as mean ± SD*.

a *Obtained from ANOVA*.

Table 3 illustrates serum levels of inflammatory biomarkers across the MD score tertiles. After controlling for age, gender, BMI, and physical activity, participants in the top tertile had significantly lower CRP (7.80 ± 1.87 vs. 37.36 ± 2.05 mg/l) and ESR (14.08 ± 2.17 vs. 42.65 ± 2.37 mm/hr.) than those in the bottom tertile (*P* < 0.001).

**Table 3:** Inflammatory biomarkers across tertiles of Mediterranean diet.

	**Tertiles of MD score**			
	**T1**	**T2**	**T3**	P^*^
	***n = 80***	***n = 81***	***n = 89***	
CRP (mg/l)	37.36 ± 2.05	15.18 ± 1.90	7.80 ± 1.87	<0.001
ESR (mm/hr)	42.65 ± 2.37	19.29 ± 2.21	14.08 ± 2.17	<0.001

*CRP, C-reactive protein; ESR, erythrocyte sedimentation rate*.

*Data are presented as mean ± SE*.

* *Values were adjusted for age, sex, BMI, and physical activity using ANCOVA*.

Crude and multivariable-adjusted odds ratios (OR), with 95% confidence intervals (CIs), for severe COVID-19 according to tertiles of MD score are presented in Table 4. Patients in the top tertile of the MD score had lower odds of having severe COVID-19 than patients in the bottom tertile, in the crude model (OR: 0.15; 95% CI: 0.08, 0.30, *P* < 0.001). After controlling for potential confounding factors, participants with the highest MD score were 84% less likely to have severe COVID-19 than those with the lowest score (OR: 0.16; 95% CI: 0.08, 0.32, *P* < 0.001). Additional adjustment for BMI attenuated, but did not eliminate, the association (OR: 0.23; 95% CI: 0.11, 0.50, P < 0.001). When the analyses were done individually based on BMI status, surprisingly the association was significant only among overweight or obese patients; implying an 86% decreased probability of having severe COVID-19 (OR: 0.14; 95% CI: 0.06, 0.36, *P* < 0.001). However, there was no association among normal weight patients (OR: 0.35; 95% CI: 0.07, 1.77, *P* = 0.22) (Supplementary Table 1). Concerning the different components of the MD pattern, we found a significant inverse association between odds of severe COVID-19 and consumption of vegetables (OR: 0.31; 95% CI: 0.15, 0.64, *P* = 0.002), fruits (OR: 0.35; 95% CI: 0.17, 0.74, *P* = 0.006), legumes (OR: 0.35; 95% CI: 0.17, 0.71, *P* = 0.004), nuts (OR: 0.35; 95% CI: 0.16, 0.74, *P* = 0.006), whole grains (OR: 0.39; 95% CI: 0.19, 0.80, *P* = 0.01), and fish (OR: 0.31; 95% CI: 0.15, 0.64, *P* = 0.002). There were no additional significant association between other MD components and severity of COVID-19 (Supplementary Table 2).

**Table 4:** Odds ratio (95% CI) of severe disease according to tertiles of Mediterranean diet.

	**Tertiles of MD score**			
	**T1**	**T2**	**T3**	P^*^
	***n = 80***	***n = 81***	***n = 89***	
Crude	1.00	0.17 (0.08, 0.33)	0.15 (0.08, 0.30)	<0.001
Model 1	1.00	0.17 (0.09, 0.35)	0.17 (0.08, 0.33)	<0.001
Model 2	1.00	0.18 (0.08, 0.37)	0.16 (0.08, 0.32)	<0.001
Model 3	1.00	0.24 (0.11, 0.51)	0.23 (0.11, 0.50)	<0.001

*BMR, Basal Metabolic Rate; BMI, body mass index*.

*Model 1: Adjusted for age, sex, and energy intake/BMR*.

*Model 2: Further adjusted for physical activity, supplement use, corticosteroids use, and antiviral drugs use*.

*Model 3: Further adjusted for BMI*.

* *Obtained from Binary logistic regression*.

Table 5 outlines the crude and multivariable-adjusted OR for COVID-19 symptoms according to tertiles of the MD score. After controlling for potential confounders, there was a significant inverse relationship between the MD score and the likelihood of experiencing COVID-19 symptoms such as dyspnea (OR: 0.32; 95% CI: 0.13, 0.76, *P* = 0.03), cough (OR: 0.11; 95% CI: 0.05, 0.26, *P* < 0.001), fever (OR: 0.11; 95% CI: 0.03, 0.37, *P* < 0.001), chilling (OR 0.06; 95% CI: 0.01, 0.26, *P* < 0.001), weakness (OR: 0.34; 95% CI: 0.15, 0.76, *P* = 0.01), myalgia (OR: 0.34; 95% CI: 0.16, 0.72, *P* = 0.005), nausea and vomiting (OR: 0.06; 95% CI: 0.01, 0.30, *P* < 0.001), and sore throat (OR: 0.08; 95% CI: 0.03, 0.21, *P* < 0.001).

**Table 5:** Odds ratio (95% CI) for symptoms of COVID-19 according to tertiles of Mediterranean diet.

	**Tertiles of MD score**			
	**T1**	**T2**	**T3**	P^*^
	***n = 80***	***n = 81***	***n = 89***	
**Dyspnea**				
Crude	1.00	0.13 (0.06, 0.28)	0.18 (0.08, 0.40)	<0.001
Model 1	1.00	0.13 (0.06, 0.29)	0.20 (0.09, 0.45)	<0.001
Model 2	1.00	0.16 (0.07, 0.36)	0.22 (0.10, 0.50)	<0.001
Model 3	1.00	0.20 (0.08, 0.47)	0.32 (0.13, 0.76)	0.03
**Cough**				
Crude	1.00	0.18 (0.08, 0.40)	0.07 (0.03, 0.16)	<0.001
Model 1	1.00	0.18 (0.08, 0.40)	0.08 (0.03, 0.17)	<0.001
Model 2	1.00	0.18 (0.08, 0.42)	0.07 (0.03, 0.17)	<0.001
Model 3	1.00	0.25 (0.11, 0.59)	0.11 (0.05, 0.26)	<0.001
**Fever**				
Crude	1.00	0.12 (0.04, 0.38)	0.09 (0.03, 0.27)	<0.001
Model 1	1.00	0.13 (0.04, 0.39)	0.09 (0.03, 0.28)	<0.001
Model 2	1.00	0.12 (0.04, 0.39)	0.10 (0.03, 0.29)	<0.001
Model 3	1.00	0.15 (0.05, 0.47)	0.11 (0.03, 0.37)	<0.001
**Chilling**				
Crude	1.00	0.06 (0.01, 0.25)	0.04 (0.01, 0.19)	<0.001
Model 1	1.00	0.06 (0.01, 0.26)	0.04 (0.01, 0.20)	<0.001
Model 2	1.00	0.06 (0.01, 0.26)	0.05 (0.01, 0.21)	<0.001
Model 3	1.00	0.07 (0.01, 0.31)	0.06 (0.01, 0.26)	<0.001
**Weakness**				
Crude	1.00	0.39 (0.20, 0.75)	0.26 (0.13, 0.52)	<0.001
Model 1	1.00	0.42 (0.21, 0.84)	0.26 (0.12, 0.53)	<0.001
Model 2	1.00	0.41 (0.20, 0.82)	0.25 (0.12, 0.53)	<0.001
Model 3	1.00	0.51 (0.24, 1.08)	0.34 (0.15, 0.76)	0.01
**Myalgia**				
Crude	1.00	0.46 (0.24, 0.86)	0.26 (0.14, 0.49)	<0.001
Model 1	1.00	0.50 (0.26, 0.96)	0.26 (0.13, 0.51)	<0.001
Model 2	1.00	0.51 (0.26, 0.99)	0.26 (0.13, 0.52)	<0.001
Model 3	1.00	0.64 (0.32, 1.30)	0.34 (0.16, 0.72)	0.005
**Nausea and vomiting**				
Crude	1.00	0.27 (0.11, 0.65)	0.06 (0.01, 0.25)	<0.001
Model 1	1.00	0.26 (0.10, 0.63)	0.05 (0.01, 0.22)	<0.001
Model 2	1.00	0.33 (0.13, 0.81)	0.05 (0.01, 0.25)	<0.001
Model 3	1.00	0.36 (0.13, 0.94)	0.06 (0.01, 0.30)	<0.001
**Sore throat**				
Crude	1.00	0.43 (0.23, 0.81)	0.07 (0.03, 0.18)	<0.001
Model 1	1.00	0.44 (0.23, 0.84)	0.06 (0.02, 0.16)	<0.001
Model 2	1.00	0.41 (0.21, 0.81)	0.06 (0.02, 0.15)	<0.001
Model 3	1.00	0.50 (0.24, 1.02)	0.08 (0.03, 0.21)	<0.001

*BMR, Basal Metabolic Rate; BMI, body mass index*.

*Model 1: Adjusted for age, sex, and energy intake/BMR*.

*Model 2: Further adjusted for physical activity, supplement use, corticosteroids use, and antiviral drugs use*.

*Model 3: Further adjusted for BMI*.

* *Obtained from Binary logistic regression*.

## Discussion

This study found an inverse association between a higher MD score and odds of having severe COVID-19 as well as a shorter duration of hospitalization and convalescence. Even though there was little difference in total energy intake among the participants, those in the upper tertile of MD scores had significantly lower levels of inflammatory biomarkers such as CRP (7.80 ± 1.87 vs. 37.36 ± 2.05 mg/l) and ESR (14.08 ± 2.17 vs. 42.65 ± 2.37) than those in the lowest tertile. Both CRP and ESR are widely used as systemic inflammatory biomarkers (32). The ESR may reveal the presence of inflammation in the body because it measures the rate at which red blood cells drop or settle in the plasma of a randomly selected anticoagulated blood sample, and inflammation can result in cell clumping (32). On the other hand, CRP is an unspecific acute-phase reactant that is produced and synthesized in the liver in response to a variety of pro-inflammatory cytokines (32).

Taking this comparison into account, those in the upper tertile of the MD consumed more protein, carbohydrates, fiber, n-3 fatty acids, vitamin B complex (B1, B2, B6, B12, folate), calcium, potassium, and magnesium, while consuming less total fat, primarily saturated fat. This is an expected result, as individuals with the highest MD scores consumed significantly more fruits, vegetables, legumes, nuts, whole grains, fish, and lean meats, all of which contain functional elements that may help mitigate COVID-related inflammation. COVID-19 causes a cytokine storm (e.g., interferon-alpha, interferon-gamma, interleukin (IL)-1, IL-6, IL-12, IL-18, IL-33, tumor necrosis factor-alpha (TNF-α), and transforming growth factor-β, a central tenet that exacerbates the severity of COVID-19 (33). RCTs demonstrated the beneficial effects of the MD on the inflammatory status and primary events. In an RCT involving 372 participants over 3 months, adequate consumption of cereals and fruits, combined with a higher intake of nuts and olive oil, was associated with lower levels of IL-6, CRP, and other inflammatory biomarkers such as vascular cell adhesion molecule-1 (VCAM-1) and intercellular adhesion molecule-1 (ICAM-1) (34, 35). Most importantly, the Prevención con Dieta Mediterránea (PREDIMED) trial, a multicenter study involving 7,447 patients at high risk of cardiovascular disease, demonstrated that the MD has long-term benefits by reducing the incidence of major cardiovascular events compared to a low-fat diet after a mean follow-up of 5 years (36–40). Concerning polyphenols, a sub-study of the PREDIMED trial involving 1,170 elderly participants showed a significant inverse association between polyphenol intake as measured by the urinary biomarker total polyphenol excretion and inflammatory markers, with a significant decrease in plasma concentrations of VCAM-1, ICAM-1, IL-6, TNF-α, and monocyte chemoattractant protein (41). The favorable effects of the MD pattern on platelet-activating factor-induced platelet aggregation is another potential against COVID-19, as the platelet-activating factor is a critical molecule in the pathogenesis of this ailment (42). The phytochemical mixture across the MD diet may be more efficient than individual food substances (43). Indeed, complex natural products target many facets involved in inflammation and thrombosis in a synergistic manner (43–45).

Apart from increased intake of functional nutrients, the lower intake of saturated fatty acids observed in the upper tertile of the MD score may contribute to a less inflammatory state, as this type of fat stimulates the production of CRP and TNF-α, as well as signaling pathways involving toll-like receptor-4 (46, 47). In light of this, replacing SFAs with the increase in MUFAs or PUFA found in nuts is a strong recommendation (47–50). In our study, the upper tertiles of the MD score not only had a lower intake of saturated fat but also MUFA consumption, which is an odd result given that the MD is high in MUFA due to the high consumption of olive oil. Nonetheless, this result is explicable by the fact that this research was conducted in Iran, a country whose population consumes a significant amount of nuts, primarily walnuts, which are a recognized source of PUFA (51–53). The consumption of nuts by participants in the upper tertile of the MD score was within the range associated with a sustained decline in low-grade inflammation. For example, acute and chronic studies demonstrated that nut consumption of between 20 and 90 g/day might reduce pro-inflammatory cytokines and improves associated outcomes (e.g., cardiovascular parameters and visceral fat) (54–56). Much of these benefits can be explained by high content of antioxidants, vitamins, and minerals such as selenium and alpha-tocopherol across nuts (57).

### Strengths and Limitations

As for strengths, this study shed lights on a nutritional strategy that could be used in place of isolate supplements in the fight against the COVID-19 pandemic. Such a result is significant when considering the global population. On the other hand, because our study was conducted on a single population (Iranians), we encourage additional multicenter research to confirm our findings. Due to the cross-sectional design of this study, which is a common limitation in nutrition and medical research, this study cannot infer causation, and thus recommendations cannot be affirmed. Despite extensive adjustment for potential confounding variables, the possibility of residual confounding cannot be completely ruled out. It should be noted that the sample size and all participants were drawn from a single-center, so generalizability to the general public should be approached with caution. Additionally, the FFQs rely on interviewees' memory, and despite our efforts to accurately measure their estimated nutrient intake over a year in grams, the memory bias inherent in this type of questionnaire cannot be ignored, particularly in relation to overweight and obese individuals, who are more likely to underreport their energy intake (58). To overcome this, we controlled for the proportion of energy intake to basal metabolic rate (BMR) in the analyses, which was calculated using Schofield equation (59). MD score calculation method is limited as it is based on the relationship between each participant's food consumption and depends on whether or not the population has a high or low rate of adherence to the MD. This study examined the effect of a dietary pattern on COVID-19 in a population with no pre-existing conditions and a low-risk profile (such as diabetes, cardiovascular disease, smoking, morbid obesity). Those who are at greater risk may experience a different response to the dietary items. Therefore, it would be prudent for future research to evaluate high-risk groups. We have no specific information on the type of dietary supplements consumed, hence we evaluate them all in a similar way. We also did not assess hospital nutrition or nutritional risk. To the extent that recent research shows that customized hospital diets have been used for COVID-19 patients (60), this merits further attention.

Finally, we did not assess a robust laboratory panel for the inflammatory status, such as the whole inflammatory signaling cascade of CRP, which includes at least IL-1β, IL-6, and TNF-α (61). Insufficient data on the use of nonsteroidal anti-inflammatory drugs (NSAIDs), monoclonal antibodies, IL-6 inhibitors, and blood pressure medications was another limitation. Furthermore, we did not assess the level of antioxidants and polyphenols in urine or blood. Therefore, additional research is necessary to better understand the metabolic pathways involved in this process.

## Conclusion

Following a MD pattern can result in a favorable profile against COVID-19, as we observed that a higher MD score was associated with shorter length of hospital stay, convalescence, symptoms, as well as reduced severity and inflammatory status (low CRP and ESR levels) in patients with COVID-19. However, RCTs are necessary in order to provide recommendations for any population.

## Data Availability Statement

The raw data supporting the conclusions of this article will be made available by the authors, without undue reservation.

## Ethics Statement

The studies involving human participants were reviewed and approved by Kashan University of Medical Sciences, IR.KAUMS.MEDNT.REC.1400.048. The patients/participants provided their written informed consent to participate in this study.

## Author Contributions

NZ and SM: conceptualization, formal analysis, writing original draft, and writing review and editing. AE, MT, HK, and CA: data collection. AM: supervision, conceptualization, methodology, investigation, funding acquisition, formal analysis, writing original draft, and writing review and editing. HS, FT, HC, and KV: writing review and editing. All authors contributed to the article and approved the submitted version.

## Conflict of Interest

The authors declare that the research was conducted in the absence of any commercial or financial relationships that could be construed as a potential conflict of interest.

## Publisher's Note

All claims expressed in this article are solely those of the authors and do not necessarily represent those of their affiliated organizations, or those of the publisher, the editors and the reviewers. Any product that may be evaluated in this article, or claim that may be made by its manufacturer, is not guaranteed or endorsed by the publisher.

## References

[1] Cascella M Rajnik M Aleem A Dulebohn SC Di Napoli R. Features, Evaluation, and Treatment of Coronavirus (COVID-19). Treasure Island, FL: StatPearls Publishing (2022). 32150360

[2] Caldera-Crespo LA Paidas MJ Roy S Schulman CI Kenyon NS Daunert S. Experimental models of COVID-19. Front Cell Infect Microbiol. (2022) 11:792584. 10.3389/fcimb.2021.792584 35096645 PMC8791197

[3] Tuczyńska M Matthews-Kozanecka M Baum E. Accessibility to non-COVID health services in the world during the COVID-19 pandemic: review. Front Public Health. (2021) 9:760795. 10.3389/fpubh.2021.760795 34976922 PMC8716399

[4] Kaye AD Okeagu CN Pham AD Silva RA Hurley JJ Arron BL. Economic impact of COVID-19 pandemic on healthcare facilities and systems: international perspectives. Best Pract Res Clin Anaesthesiol. (2021) 35:293–306. 10.1016/j.bpa.2020.11.009 34511220 PMC7670225

[5] Palacios Cruz M Santos E Velázquez Cervantes MA León Juárez M. COVID-19, a worldwide public health emergency. Rev Clin Esp. (2021) 221:55–61. 10.1016/j.rceng.2020.03.001 33998479 PMC7173827

[6] Nalbandian A Sehgal K Gupta A Madhavan MV McGroder C Stevens JS. Post-acute COVID-19 syndrome. Nat Med. (2021) 27:601–15. 10.1038/s41591-021-01283-z 33753937 PMC8893149

[7] Peramo-Álvarez FP López-Zúñiga M López-Ruz M. Medical sequels of COVID-19. Med Clin. (2021) 157:388–94. 10.1016/j.medcle.2021.04.008 34632064 PMC8491931

[8] Logue JK Franko NM McCulloch DJ McDonald D Magedson A Wolf CR. Sequelae in adults at 6 months after COVID-19 infection. JAMA Network Open. (2021) 4:e210830-e. 10.1001/jamanetworkopen.2021.0830 PMC7896197 33606031

[9] Rando HM Wellhausen N Ghosh S Lee AJ Dattoli AA Hu F. Identification and development of therapeutics for COVID-19. Msystems. (2021) 6:e00233–21. 10.1128/mSystems.00233-21 34726496 PMC8562484

[10] Santos HO Tinsley GM da Silva GA Bueno AA. Pharmaconutrition in the clinical management of COVID-19: a lack of evidence-based research but clues to personalized prescription. J Pers Med. (2020) 10:145. 10.3390/jpm10040145 32992693 PMC7712662

[11] Antwi J Appiah B Oluwakuse B Abu BAZ. The nutrition-COVID-19 interplay: a review. Curr Nutr Rep. (2021) 10:364–74. 10.1007/s13668-021-00380-2 34837637 PMC8627159

[12] Sinha P Matthay MA Calfee CS. Is a “Cytokine Storm” relevant to COVID-19? JAMA Intern Med. (2020) 180:1152–4. 10.1001/jamainternmed.2020.3313 32602883

[13] Hu B Huang S Yin L. The cytokine storm and COVID-19. J Med Virol. (2021) 93:250–6. 10.1002/jmv.26232 32592501 PMC7361342

[14] Al-Okbi SY. Nutraceuticals of anti-inflammatory activity as complementary therapy for rheumatoid arthritis. Toxicol Ind Health. (2014) 30:738–49. 10.1177/0748233712462468 23104728

[15] Mousavi SM Djafarian K Mojtahed A Varkaneh HK Shab-Bidar S. The effect of zinc supplementation on plasma C-reactive protein concentrations: A systematic review and meta-analysis of randomized controlled trials. Eur J Pharmacol. (2018) 834:10–6. 10.1016/j.ejphar.2018.07.019 30012497

[16] Zargarzadeh N Severo JS Pizarro AB Persad E Mousavi SM. The effects of folic acid supplementation on pro-inflammatory mediators: a systematic review and dose–response meta-analysis of randomized controlled trials. Clin Ther. (2021) 43:e346–e63. 10.1016/j.clinthera.2021.10.002 34857394

[17] Thomas S Patel D Bittel B Wolski K Wang Q Kumar A. Effect of high-dose zinc and ascorbic acid supplementation vs usual care on symptom length and reduction among ambulatory patients with SARS-CoV-2 infection: the COVID A to Z randomized clinical trial. JAMA Network Open. (2021) 4:e210369-e. 10.1001/jamanetworkopen.2021.0369 PMC7881357 33576820

[18] Murai IH Fernandes AL Sales LP Pinto AJ Goessler KF Duran CSC. Effect of a single high dose of Vitamin D3 on hospital length of stay in patients with moderate to severe COVID-19: a randomized clinical trial. JAMA. (2021) 325:1053–60. 10.1001/jama.2020.26848 33595634 PMC7890452

[19] Ruiz-Roso MB Knott-Torcal C Matilla-Escalante DC Garcimartín A Sampedro-Nuñez MA Dávalos A. COVID-19 lockdown and changes of the dietary pattern and physical activity habits in a cohort of patients with type 2 diabetes mellitus. Nutrients. (2020) 12:2327. 10.3390/nu12082327 32759636 PMC7468739

[20] Kim H Rebholz CM Hegde S LaFiura C Raghavan M Lloyd JF. Plant-based diets, pescatarian diets and COVID-19 severity: a population-based case–control study in six countries. BMJ Nutr Prev Health. (2021) 4:257. 10.1136/bmjnph-2021-000272 34308134 PMC8219480

[21] Hou Y-C Su W-L Chao Y-C. COVID-19 illness severity in the elderly in relation to vegetarian and non-vegetarian diets: a single-center experience. Front Nutr. (2022) 9:837458. 10.3389/fnut.2022.837458 35571931 PMC9101048

[22] Ferro Y Pujia R Maurotti S Boragina G Mirarchi A Gnagnarella P. Mediterranean diet a potential strategy against SARS-CoV-2 infection: a narrative review. Medicina. (2021) 57:1389. 10.3390/medicina57121389 34946334 PMC8704657

[23] Zabetakis I Lordan R Norton C Tsoupras A. COVID-19: The inflammation link and the role of nutrition in potential mitigation. Nutrients. (2020) 12:1466. 10.3390/nu12051466 32438620 PMC7284818

[24] Ling V Zabetakis I. The role of an anti-inflammatory diet in conjunction to COVID-19. Diseases. (2021) 9:76. 10.3390/diseases9040076 34842636 PMC8628803

[25] Hidalgo-Mora JJ García-Vigara A Sánchez-Sánchez ML García-Pérez M-Á Tarín J Cano A. The mediterranean diet: a historical perspective on food for health. Maturitas. (2020) 132:65–9. 10.1016/j.maturitas.2019.12.002 31883665

[26] Andrade V Jorge R García-Conesa M-T Philippou E Massaro M Chervenkov M. Mediterranean diet adherence and subjective well-being in a sample of Portuguese adults. Nutrients. (2020) 12:3837. 10.3390/nu12123837 33339084 PMC7765516

[27] Gotsis E Anagnostis P Mariolis A Vlachou A Katsiki N Karagiannis A. Health benefits of the Mediterranean diet: an update of research over the last 5 years. Angiology. (2015) 66:304–18. 10.1177/0003319714532169 24778424

[28] Angelidi AM Kokkinos A Katechaki E Ros E Mantzoros CS. Mediterranean diet as a nutritional approach for COVID-19. Metabolism. (2021) 114:154407. 10.1016/j.metabol.2020.154407 33080270 PMC7833284

[29] Mirmiran P Esfahani FH Mehrabi Y Hedayati M Azizi F. Reliability and relative validity of an FFQ for nutrients in the Tehran lipid and glucose study. Public Health Nutr. (2010) 13:654–62. 10.1017/S1368980009991698 19807937

[30] Trichopoulou A Costacou T Bamia C Trichopoulos D. Adherence to a Mediterranean diet and survival in a Greek population. N Engl J Med. (2003) 348:2599–608. 10.1056/NEJMoa025039 12826634

[31] Clinical Spectrum of SARS-CoV-2 Infection. (2021). Available online at: http://www.covid19treatmentguidelines.nih.gov/overview/clinical-spectrum (accessed October 19, 2021).

[32] Bray C Bell LN Liang H Haykal R Kaiksow F Mazza JJ. Erythrocyte sedimentation rate and C-reactive protein measurements and their relevance in clinical medicine. WMJ. (2016) 115:317–21. 29094869

[33] Ye Q Wang B Mao J. The pathogenesis and treatment of the ‘Cytokine Storm' in COVID-19. J Infect. (2020) 80:607–13. 10.1016/j.jinf.2020.03.037 32283152 PMC7194613

[34] Fitó M Guxens M Corella D Sáez G Estruch R de la Torre R. Effect of a traditional Mediterranean diet on lipoprotein oxidation: a randomized controlled trial. Arch Intern Med. (2007) 167:1195–203. 10.1001/archinte.167.11.1195 17563030

[35] Urpi-Sarda M Casas R Chiva-Blanch G Romero-Mamani ES Valderas-Martínez P Arranz S. Virgin olive oil and nuts as key foods of the Mediterranean diet effects on inflammatory biomakers related to atherosclerosis. Pharmacol Res. (2012) 65:577–83. 10.1016/j.phrs.2012.03.006 22449789

[36] Martínez-González MA Salas-Salvadó J Estruch R Corella D Fitó M Ros E. Benefits of the mediterranean diet: insights from the PREDIMED study. Prog Cardiovasc Dis. (2015) 58:50–60. 10.1016/j.pcad.2015.04.003 25940230

[37] Guasch-Ferré M Salas-Salvadó J Ros E Estruch R Corella D Fitó M. The PREDIMED trial, Mediterranean diet and health outcomes: how strong is the evidence? Nutr Metab Cardiovasc Dis. (2017) 27:624–32. 10.1016/j.numecd.2017.05.004 28684083

[38] Estruch R Ros E Salas-Salvadó J Covas MI Corella D Arós F. Primary prevention of cardiovascular disease with a mediterranean diet supplemented with extra-virgin olive oil or nuts. N Engl J Med. (2018) 378:e34. 10.1056/NEJMoa1800389 29897866

[39] Billingsley HE Carbone S. The antioxidant potential of the Mediterranean diet in patients at high cardiovascular risk: an in-depth review of the PREDIMED. Nutr Diabetes. (2018) 8:13. 10.1038/s41387-018-0025-1 29549354 PMC5856841

[40] Estruch R Martínez-González MA Corella D Salas-Salvadó J Ruiz-Gutiérrez V Covas MI. Effects of a Mediterranean-style diet on cardiovascular risk factors: a randomized trial. Ann Intern Med. (2006) 145:1–11. 10.7326/0003-4819-145-1-200607040-00004 16818923

[41] Medina-Remón A Casas R Tressserra-Rimbau A Ros E Martínez-González MA Fitó M. Polyphenol intake from a Mediterranean diet decreases inflammatory biomarkers related to atherosclerosis: a substudy of the PREDIMED trial. Br J Clin Pharmacol. (2017) 83:114–28. 10.1111/bcp.12986 27100393 PMC5338147

[42] Detopoulou P Demopoulos CA Antonopoulou S. Micronutrients, phytochemicals and Mediterranean diet: a potential protective role against COVID-19 through Modulation of PAF actions and metabolism. Nutrients. (2021) 13:462. 10.3390/nu13020462 33573169 PMC7911163

[43] Panossian A Brendler T. The role of adaptogens in prophylaxis and treatment of viral respiratory infections. Pharmaceuticals. (2020) 13:236. 10.3390/ph13090236 32911682 PMC7558817

[44] Santos HO Genario R Gomes GK Schoenfeld BJ. Cherry intake as a dietary strategy in sport and diseases: a review of clinical applicability and mechanisms of action. Crit Rev Food Sci Nutr. (2020) 1–14. 10.1080/10408398.2020.1734912 32126807

[45] Wang P Zhang Q Hou H Liu Z Wang L Rasekhmagham R. The effects of pomegranate supplementation on biomarkers of inflammation and endothelial dysfunction: A meta-analysis and systematic review. Complement Ther Med. (2020) 49:102358. 32147056 10.1016/j.ctim.2020.102358

[46] Lee JY Sohn KH Rhee SH Hwang D. Saturated fatty acids, but not unsaturated fatty acids, induce the expression of cyclooxygenase-2 mediated through toll-like receptor 4. J Biol Chem. (2001) 276:16683–9. 10.1074/jbc.M011695200 11278967

[47] Fritsche KL. The science of fatty acids and inflammation. Adv Nutr. (2015) 6:293s−301s. 10.3945/an.114.006940 PMC4424767 25979502

[48] Ruiz-Núñez B Dijck-Brouwer DA Muskiet FA. The relation of saturated fatty acids with low-grade inflammation and cardiovascular disease. J Nutr Biochem. (2016) 36:1–20. 10.1016/j.jnutbio.2015.12.007 27692243

[49] Kris-Etherton PM Krauss RM. Public health guidelines should recommend reducing saturated fat consumption as much as possible: YES. Am J Clin Nutr. (2020) 112:13–8. 10.1093/ajcn/nqaa110 32491173

[50] de Souza RGM Schincaglia RM Pimentel GD Mota JF. Nuts and human health outcomes: a systematic review. Nutrients. (2017) 9:1311. 10.3390/nu9121311 29207471 PMC5748761

[51] Tresserra-Rimbau A Medina-Remón A Pérez-Jiménez J Martínez-González MA Covas MI Corella D. Dietary intake and major food sources of polyphenols in a Spanish population at high cardiovascular risk: the PREDIMED study. Nutr Metab Cardiovasc Dis. (2013) 23:953–9. 10.1016/j.numecd.2012.10.008 23332727

[52] Sadighara P Ghanati K. The aflatoxin B1 content of peanut-based foods in Iran: a systematic review. Rev Environ Health. (2021) 37:29–33. 10.1515/reveh-2021-0065 34332516

[53] Salas-Salvadó J Bulló M Pérez-Heras A Ros E. Dietary fibre, nuts and cardiovascular diseases. Br J Nutr. (2006) 96:S45–51. 10.1017/BJN20061863 17125533

[54] Lozano A Perez-Martinez P Marin C Tinahones FJ Delgado-Lista J Cruz-Teno C. An acute intake of a walnut-enriched meal improves postprandial adiponectin response in healthy young adults. Nutr Res. (2013) 33:1012–8. 10.1016/j.nutres.2013.08.010 24267040

[55] Canales A Sánchez-Muniz FJ Bastida S Librelotto J Nus M Corella D. Effect of walnut-enriched meat on the relationship between VCAM, ICAM, and LTB4 levels and PON-1 activity in ApoA4 360 and PON-1 allele carriers at increased cardiovascular risk. Eur J Clin Nutr. (2011) 65:703–10. 10.1038/ejcn.2011.20 21407247

[56] Aronis KN Vamvini MT Chamberland JP Sweeney LL Brennan AM Magkos F. Short-term walnut consumption increases circulating total adiponectin and apolipoprotein A concentrations, but does not affect markers of inflammation or vascular injury in obese humans with the metabolic syndrome: data from a double-blinded, randomized, placebo-controlled study. Metabolism. (2012) 61:577–82. 10.1016/j.metabol.2011.09.008 22075273 PMC3645917

[57] Lorenzon Dos Santos J Quadros AS Weschenfelder C Garofallo SB Marcadenti A. Oxidative stress biomarkers, nut-related antioxidants, and cardiovascular disease. Nutrients. (2020) 12:682. 10.3390/nu12030682 32138220 PMC7146201

[58] Dahle JH Ostendorf DM Zaman A Pan Z Melanson EL Catenacci VA. Underreporting of energy intake in weight loss maintainers. Am J Clin Nutr. (2021) 114:257–66. 10.1093/ajcn/nqab012 33742193 PMC8246606

[59] Schofield WN. Predicting basal metabolic rate, new standards and review of previous work. Hum Nutr Clin Nutr. (1985) 39:5–41. 4044297

[60] Detopoulou P Al-Khelefawi ZH Kalonarchi G Papamikos V. Formulation of the menu of a general hospital after its conversion to a “COVID Hospital”: a nutrient analysis of 28-day menus. Front Nutr. (2022) 9:833628. 10.3389/fnut.2022.833628 35495923 PMC9043649

[61] Slaats J Ten Oever J van de Veerdonk FL Netea MG. IL-1β/IL-6/CRP and IL-18/ferritin: distinct inflammatory programs in infections. PLoS Pathog. (2016) 12:e1005973. 10.1371/journal.ppat.1005973 27977798 PMC5158075

