# Supplementary material for: Higher Adherence to the Mediterranean Dietary Pattern Is Inversely Associated With Severity of COVID-19 and Related Symptoms: A Cross-Sectional Study
Source: Front Med (Lausanne). 2022 Jul 19;9:911273. doi: 10.3389/fmed.2022.911273 (PMC9343686; doi:10.3389/fmed.2022.911273)
Supplement: Supplementary file 1 [file Table_1.DOCX]

| **Supplementary Table 1**. Odds ratio (95% CI) of severe COVID-19 disease according to tertiles of Mediterranean diet stratified by BMI status | | | | |
| --- | --- | --- | --- | --- |
|  | Tertiles of MD score | | | |
|  | T1 | T2 | T3 | *P^*^* |
| **Normal-weight patients (BMI<25 kg/m^2^)** | | | | |
| No. of patients | 9 | 33 | 42 |  |
| Crude | 1.00 | 0.35 (0.07, 1.50) | 0.25 (0.05, 1.11) | 0.09 |
| Model 1 | 1.00 | 0.42 (0.08, 2.00) | 0.34 (0.07, 1.66) | 0.23 |
| Model 2 | 1.00 | 0.49 (0.09, 2.50) | 0.35 (0.07, 1.77) | 0.22 |
| **Overweight or obese patients (BMI≥25 kg/m^2^)** | | | | |
| No. of patients | 71 | 48 | 47 |  |
| Crude | 1.00 | 0.16 (0.07, 0.36) | 0.18 (0.08, 0.40) | <0.001 |
| Model 1 | 1.00 | 0.16 (0.07, 0.37) | 0.18 (0.08, 0.41) | <0.001 |
| Model 2 | 1.00 | 0.14 (0.06, 0.35) | 0.14 (0.06, 0.36) | <0.001 |
| Model 1: Adjusted for age, sex, and energy intake.  Model 2: Further adjusted for physical activity, supplement use, corticosteroids use, and antiviral drugs use.  * Obtained from Binary logistic regression | | | | |

| **Supplementary Table 2**. Odds ratio (95% CI) of severe COVID-19 disease according to tertiles of Mediterranean diet components | | | | |
| --- | --- | --- | --- | --- |
|  | Tertiles of MD score | | | |
|  | T1 | T2 | T3 | *P^*^* |
| **Vegetables** | | | | |
| Crude | 1.00 | 0.27 (0.14, 0.51) | 0.28 (0.15, 0.53) | <0.001 |
| Adjusted ^a^ | 1.00 | 0.28 (0.14, 0.60) | 0.31 (0.15, 0.64) | 0.002 |
| **Fruits** | | | | |
| Crude | 1.00 | 0.34 (0.18, 0.63) | 0.23 (0.12, 0.45) | <0.001 |
| Adjusted ^a^ | 1.00 | 0.43 (0.21, 0.89) | 0.35 (0.17, 0.74) | 0.006 |
| **Legumes** | | | | |
| Crude | 1.00 | 0.39 (0.21, 0.73) | 0.27 (0.14, 0.50) | <0.001 |
| Adjusted ^a^ | 1.00 | 0.44 (0.22, 0.89) | 0.35 (0.17, 0.71) | 0.004 |
| **Nuts** | | | | |
| Crude | 1.00 | 0.65 (0.35, 1.19) | 0.36 (0.19, 0.67) | 0.002 |
| Adjusted ^a^ | 1.00 | 0.42 (0.20, 0.88) | 0.35 (0.16, 0.74) | 0.006 |
| **Whole grains** | | | | |
| Crude | 1.00 | 0.82 (0.44, 1.51) | 0.39 (0.21, 0.74) | 0.004 |
| Adjusted ^a^ | 1.00 | 0.81 (0.41, 1.61) | 0.39 (0.19, 0.80) | 0.01 |
| **MUFA/SFA** | | | | |
| Crude | 1.00 | 0.70 (0.38, 1.28) | 0.71 (0.39, 1.31) | 0.27 |
| Adjusted ^a^ | 1.00 | 0.80 (0.39, 1.63) | 0.72 (0.36, 1.44) | 0.36 |
| **Meats** |  |  |  |  |
| Crude | 1.00 | 0.80 (0.43, 1.44) | 1.21 (0.65, 2.23) | 0.56 |
| Adjusted ^a^ | 1.00 | 0.68 (0.33, 1.38) | 1.05 (0.50, 2.22) | 0.87 |
| **Dairy** |  |  |  |  |
| Crude | 1.00 | 0.66 (0.36, 1.22) | 0.65 (0.35, 1.19) | 0.16 |
| Adjusted ^a^ | 1.00 | 0.94 (0.46, 1.92) | 0.82 (0.40, 1.68) | 0.58 |
| **Fish** |  |  |  |  |
| Crude | 1.00 | 0.66 (0.36, 1.21) | 0.29 (0.15, 0.56) | <0.001 |
| Adjusted ^a^ | 1.00 | 0.61 (0.30, 1.22) | 0.31 (0.15, 0.64) | 0.002 |
| ^a^ Adjusted for age, sex, and energy intake, physical activity, supplement use, corticosteroids use, and antiviral drugs use, and BMI  * Obtained from Binary logistic regression | | | | |
